# Association between Generalized Anxiety Disorder and Premenstrual Dysphoric Disorder in a Diagnostic Interviewing Study

**DOI:** 10.3390/ijerph17030988

**Published:** 2020-02-05

**Authors:** Ju-Yu Yen, Pai-Cheng Lin, Mei-Feng Huang, Wei-Po Chou, Cheng-Yu Long, Chih-Hung Ko

**Affiliations:** 1 Department of Psychiatry, Kaohsiung Municipal Ta-Tung Hospital, Kaohsiung Medical University, Kaohsiung City 80708, Taiwan; yenjuyu@cc.kmu.edu.tw (J.-Y.Y.); 1000406@ms.kmuh.org.tw (P.-C.L.); 2 Department of Psychiatry, Faculty of Medicine, College of Medicine, Kaohsiung Medical University, Kaohsiung City 80708, Taiwan; 3 Department of Psychiatry, Kaohsiung Medical University Hospital, Kaohsiung City 80708, Taiwan; 1010236@kmuh.org.tw (M.-F.H.); 1010341@kmuh.org.tw (W.-P.C.); 4 Department of Obstetrics and Gynecology, Kaohsiung Municipal Siaogang Hospital, Kaohsiung Medical University, Kaohsiung City 80708, Taiwan; 830263@kmhk.org.tw; 5 Department of Psychiatry, Kaohsiung Municipal Siaogang Hospital, Kaohsiung Medical University, Kaohsiung City 80708, Taiwan; 6 Substance and Behavior Addiction Research Center, Kaohsiung Medical University, Kaohsiung City 80708, Taiwan

**Keywords:** premenstrual dysphoric disorder, generalized anxiety disorder, depression, irritability, behavior inhibition

## Abstract

Background: Premenstrual dysphoric disorder (PMDD) demonstrates predictable, cyclic, affective and somatic symptoms that are aggravated in the late luteal phase and are resolved by menstruation. Generalized anxiety disorder (GAD) is characterized by excessive and persistant worry. The present study aims to evaluate the association between PMDD and GAD. The fluctuations of behavior inhibition, anxiety, depression, and irritability were also evaluated during the menstrual cycle among women with PMDD and healthy women. **Methods**: There were 100 women diagnosed with PMDD based on a psychiatric interview and on a prospective evaluation in three menstrual cycles. A total of 96 healthy women were recruited as controls. Each individual’s GAD diagnosis, behavior inhibition, behavior activation, depression, anxiety, and irritability were assessed in both luteal and follicular phases. **Results**: The odds ratio of women with GAD having PMDD was 7.65 (95% CI: 1.69–34.63) in relation to those without it. This association was partially mediated by behavior inhibition and irritability and was completely mediated by depression. Women with PMDD and GAD had higher anxiety during the luteal phase and higher PMDD severity, depression, and irritability than those without GAD in the follicular phase. There is no difference in anxiety, depression, or irritability between the luteal and follicular phases among women with PMDD and GAD. **Conclusions**: Women with GAD were more likely to have PMDD. Anxiety, depression, and irritability symptoms in women with PMDD and GAD were not relieved in the follicular phase. Thus, GAD should be assessed for women with PMDD. Their anxiety, depression, and irritability should be intervened not only in the luteal phase, but also in the follicular phase. Depression, irritability and behavior inhibition mediated the association between PMDD and GAD. Intervening with these mediators to attenuate GAD and PMDD comorbidity should be researched in the future.

## 1. Introduction

Premenstrual dysphoric disorder (PMDD) is a depressive disorder involving cyclic psychological, cognitive, and somatic symptoms, leading to functional impairment within the late luteal phase of the menstrual cycle [1]. Since the scientific information on the diagnosis, treatment, and validation of PMDD is advanced sufficiently, it is recruited as a depressive disorder in the *Diagnostic and Statistical Manual of Mental Disorders*, *Fifth Edition* (*DSM*-*5*) [2,3]. PMDD is associated with anxiety and depressive disorder [4,5]. However, how women with comorbidities exhibit symptoms during the menstrual cycle has not yet been completely understood.

### 1.1. Comorbidity of Anxiety with PMDD

Halbreich suggested that altered sensitivity to gonadal hormone fluctuations, such as decline of estrogen or progesterone in the late luteal phase and during the menstrual cycle, may contribute to premenstrual symptoms in vulnerable women [6]. Although studies have not revealed any significant correlation between anxiety symptoms and estrogen or progesterone levels [7], our previous results demonstrated that anxiety symptoms were exacerbated in the late luteal phase and attenuated in the follicular phase [8]. Halbreich [9] suggested that PMDD could share anxiety vulnerability with an anxiety disorder, such as generalized anxiety disorder (GAD) or panic disorder. For example, lactate infusion [10] and CO_2_ challenge [11] exaggerate panic symptoms among women with PMDD, particularly in the late luteal phase. Breaux, Hartlage, and Gehlert [12] and Kim et al. [13] have claimed that reviews of the literature revealed that 4%–38% of PMDD in a clinical setting had comorbidity with GAD. Further, Kim et al. also suggested a physiological overlap between PMDD and anxiety disorders [13]. These studies and reviews might support an association between PMDD and GAD.

### 1.2. Generalized Anxiety Disorder

Generalized anxiety disorder (GAD) is characterized by excessive and persistent worry and stress that are difficult to control and often accompanied by insomnia, restlessness, muscle tension, and concentration problems. Symptoms occur more days than not for at least 6 months, according to the DSM-5 [3]. GAD is one of the most common mental disorders in both the community and in primary care facilities [14], with 4%–7% lifetime prevalence and 1%–4% annual incidence [15,16]. Comorbidity with major depressive or other anxiety disorders is commonly observed in patients with GAD and is associated with a poorer prognosis compared with isolated GAD [17]. Adewuya et al. [4] reported a higher rate of GAD (16%) among women with PMDD in an epidemiological study among university students. The co-morbidity between GAD and PMDD had also been supported by literature review of Kim et al. [13]. More evidence is required to understand the association between PMDD and GAD and the factors contributing to their comorbidity.

According to the study of Kornstein et al. [18], women with major depressive disorder exacerbate their depression in the premenstrual phase. Cook et al. and van Veen et al. demonstrated the premenstrual exacerbation of anxiety symptoms among women with panic disorder [19] and social anxiety disorder [20], respectively. GAD is characterized by a long-term anxiety symptom that occurs more days than not. However, how PMDD women with GAD present their emotional symptoms (such as depression or irritability) has not yet been evaluated. Moreover, the behavioral inhibition system (BIS) and the behavioral approach system (BAS) represent the behavior reactions to stimuli that signal conditioned aversive and rewarding responses, respectively [21]. The BIS system might represent sensitivity to aversion and vulnerability to anxiety symptoms [22]. Both PMDD [23] and GAD [24] are associated with higher BIS scores. BIS could be a shared characteristic between PMDD and GAD and deserve further evaluation.

On the basis of the aforementioned studies, we hypothesize that GAD is more prevalent among women with PMDD than those without. PMDD women with GAD have worse premenstrual symptoms than those without GAD. Furthermore, because GAD is a chronic disorder, emotional symptoms, such as depression or irritability, persist even in the follicular phase among women with PMDD. Lastly, the BIS mediates the association between PMDD and GAD. Therefore, the present study investigates (1) the association between PMDD and GAD; (2) BIS, BAS, depression, irritability, and anxiety in women with PMDD; (3) the premenstrual change in PMDD symptoms, anxiety, depression, and irritability among women with PMDD with and without GAD; and (4) the role of the behavioral inhibition system (BIS), depression, and irritability in the association between PMDD and GAD.

## 2. Methods

### 2.1. Participants

This study was approved by the Institutional Review Board of Kaohsiung Medical University Hospital (KMUHIRB-20130004). Participants with untreated PMDD and healthy women were recruited using an advertisement posted on a university campus from December 2013–April 2015. Participants in the PMDD group were required to have five or more of the 11 symptoms in the DSM-5 criteria for PMDD [3] that relieved in 2–3 days after the onset of menstruation. Healthy women in the control group had a maximum of two of the 11 symptoms among these criteria or no significant impairment with mild symptoms. Individuals currently using psychotropic and gonadotropic medication were excluded from the study to prevent their effects on serotonin and estrogen function. Those with a history of bipolar I disorder and psychotic disorder were also excluded based on the Mini-International Neuropsychiatric Interview (MINI) [25]. Both PMDD and the control group were interviewed by 1 of 2 psychiatrists to confirm their diagnosis with GAD and PMDD based on the DSM-5 criteria [3]. Consequently, 137 participants in the PMDD group and 96 controls were diagnosed as having and not having PMDD, respectively. All participants were traced in the three-menstrual-cycle prospective study after providing informed consent. A total of 100 women with PMDD and 96 controls fulfilled all the recruitment criteria and entered the final analysis.

### 2.2. Measures

**DSM-5 PMDD diagnostic criteria** [3]: In most menstrual cycles within the preceding year, patients with PMDD have at least five symptoms, among which are marked affective lability, irritability, depressed mood, anxiety, inability to concentrate, lethargy, overeating, sleep problems, a sense of being overwhelmed, and physical symptoms occurring during the final week before the onset of menses and starting to remit within a few days after menstruation; these symptoms are minor or absent in the week postmenses. The symptoms are associated with clinically significant distress related to work, school, social activities, or relationships confirmed on the basis of prospective ratings during at least two symptomatic cycles. The prospective ratings are discussed in Section 2.3.

**The Chinese version of the MINI** [25]: The MINI is a structured diagnostic interview, developed jointly by psychiatrists and clinicians in the United States and Europe, for psychiatric disorders in *DSM Fourth Edition*, *Text Revision* [26]. We also conducted a diagnostic interview based on the GAD modules by using the Chinese version of the MINI to determine the existence of GAD.

**PMDD severity questionnaire***:* The PMDD severity questionnaire (PMDDSQ) was developed to assess the severity of PMDD symptoms over the menstrual cycle. The questionnaire includes an 11-point Likert scale for rating the severity of the 11 PMDD criteria in the *DSM Fourth Edition*, *Text Revision* [26]. Every symptom, such as “Depressed mood” or “Irritability/anger/argument with others”, was rated from 0 (no symptoms) to 10 (extremely severe symptoms). Participants were asked to respond to the questions based on the symptoms experienced at the time the questionnaire was completed. The total score of the 11 items was used to represent severity. The questionnaire had a Cronbach’s alpha of 0.98, and the four-week test–retest reliability was 0.92 [27].

**BIS and BAS Scales***:* The BIS and BAS scales were a 4-point Likert scale and were designed to assess individual differences in the sensitivity of the two motivational systems proposed by Gray [21]. The BIS scale consists of 7 items and measures the degree to which respondents expect to feel anxiety when confronted with cues for punishment, such as “I feel worried when I think I have done poorly at something”. The BAS scale includes subscales of reward responsiveness (four items), drive (four items), and fun seeking (five items) that measure the degree to which rewards lead to positive emotions, a person’s tendency to actively pursue appetitive goals, and the tendency to seek out and impulsively engage in potentially rewarding activities, respectively. The test–retest reliability was 0.66 for BIS, 0.59 for reward responsiveness, 0.66 for drive, and 0.69 for fun seeking [28].

**The Center for Epidemiological Studies**’ **Depression Scale (CES-D)**: The CESD is a 20-item self-administered questionnaire, such as “I felt depressed”, assessing participants’ frequency of depressive symptoms over the last week [29,30]. The Cronbach’s alpha of CES-D in the present study was 0.93. A higher score indicates higher depression in this study.

**The Penn state worry questionnaire** (**PSWQ**): The PSWQ is a 16-item, five-point Likert self-administered scale for anxiety symptoms, such as “My worries overwhelm me”. It significantly discriminates college samples who met all, some, or none of the DSM-III-R diagnostic criteria for generalized anxiety disorder [31]. The Cronbach’s alpha of PSWQ in the present study was 0.92. A higher score indicates higher anxiety in this study.

**The Buss–Durkee Hostility Inventory**-**Chinese Version**-**Short Form** (BDHIC-SF): The 20-item, five-point Likert-type BDHIC-SF was used to assess the intensity of the irritability, such as “When frustrated, I let my irritation show” [32]. The Cronbach’s alpha of BDHIC-SF in the present study was 0.90. A higher score indicates higher irritability in this study.

### 2.3. Procedures

All participants were assessed in both the follicular phase (7–14 days after the last menstruation) and premenstrual phase (within 1 week before menstruation), predicted according to their last menstruation cycle. Half of the participants (69 women with PMDD and 49 controls) were evaluated first in the late luteal phase and again in the follicular phase to complete the PMDDSQ, BIS and BAS scale, CESD, PSWQ, and BDHIC-SF. The other participants (68 women with PMDD and 47 controls) were evaluated in the reverse order. After the aforementioned investigation in the first menstrual cycle, all participants completed the PMDDSQ weekly for the next two menstrual cycles. The questionnaire was sent to participants in an e-mail and was required to be completed within a given duration. The PMDD symptoms are defined to aggravated in the late luteal phase and to be resolved by menstruation [3]. Referring to the previously reported criteria of the PMDD symptomatic cycle [33], scores in the late luteal phase should be 30% higher than the lowest individual scores in the menstrual cycle. Participants diagnosed with PMDD were required to fulfill these criteria for two consecutive menstrual cycles to be categorized in the PMDD group.

### 2.4. Statistical Analyses

First, we compared age, education level, severity of PMDD, behavior inhibition, depression, and irritability during the premenstrual and follicular phases between the PMDD and control groups by using an independent *t* test. A chi-square analysis was used to evaluate the association between GAD and PMDD. The odds ratio of GAD in the PMDD group relative to the control group was determined using a logistic regression after controlling for age. Then, we regressed PMDD on GAD and its associated factors, such as BIS, depression, and irritability, to test their mediating effect on the association between GAD and PMDD [34]. Then, differences in PMDD severity, behavior inhibition, anxiety, depression, and irritability between women with PMDD, with and without GAD, were tested using an independent *t* test. Finally, the difference in PMDD severity, anxiety, and irritability between the follicular and luteal phases was evaluated using a paired *t* test for women with PMDD with and without GAD. A *p* value of <0.05 was considered statistically significant for all analyses.

## 3. Results

A total of 37 symptomatic women were excluded from the PMDD group because they did not fulfill the criterion of two consecutive symptomatic cycles [33] in the prospective investigation conducted using PMDDSQ. A total of 100 women with PMDD and 96 controls fulfilled the recruitment criteria. The independent *t* test revealed no significant difference in age or education level between the PMDD and control groups (Table 1).

### 3.1. BIS, Depression, and Irritability with PMDD

The independent *t* test demonstrated that women with PMDD had higher BIS, depression, and irritability scores in both the luteal and follicular phases than the controls. Furthermore, the paired *t* test demonstrated that BIS, depression, and irritability scores were higher in the luteal phase than in the follicular phase in the PMDD group, but not among controls. This result demonstrated the premenstrual exacerbation of BIS, depression, and irritability.

### 3.2. Association between GAD and PMDD when Controlling for BIS, Depression, or Irritability

A chi-square analysis demonstrated that women with GAD were more likely to have PMDD. A logistic regression demonstrated that the odds of women with GAD having PMDD were 7.65 times greater (95% CI: 1.69–34.63; Table 2, Model 1) than that of women without GAD, when controlling for age. After controlling for BIS or irritability, the odds of women with GAD having PMDD were 5.22 (95% CI: 1.11–24.54; Table 2, Model 2) and 4.96 (95% CI: 1.04–23.60; Table 2, Model 3) times greater, respectively. When controlling for depression, the association of GAD with PMDD was nonsignificant (Table 2, Model 4).

### 3.3. Severity of PMDD, BIS, Anxiety, Depression, and Irritability in GAD among Women with PMDD

The independent *t* test demonstrated that women with GAD had higher luteal anxiety than those without it among the PMDD group. Furthermore, they had higher follicular PMDD severity, anxiety, depression, and irritability than those without GAD. The paired *t* test demonstrated that women with PMDD and GAD had significantly lower PMDD severity and BIS in the follicular phase than in the luteal phase. However, no difference in anxiety, depression, or irritability was observed between the follicular and luteal phases among women with PMDD and GAD. Furthermore, women with PMDD but without GAD had significantly lower scores in follicular PMDD severity, BIS, anxiety, depression, and irritability than those in the luteal phase.

## 4. Discussion

### 4.1. Comorbidity of GAD in PMDD

This is the first case-control study to demonstrate that women with PMDD had a higher comorbidity of GAD than those without. Because women with PMDD have biological markers of anxiety vulnerability [11] and cognitive anxiety sensitivity [35], 53% of women seeking treatment for PMDD fulfilled the diagnostic criteria for an anxiety disorder (primarily phobias or GAD) [36]. Our data demonstrated that 14% of women with PMDD in the cohort had comorbid GAD. This comorbidity rate was similar to data from Adewuya et al. [4] (16%). These results support the conclusion that women with PMDD have a higher GAD comorbidity rate than the general population does; the rate was 3.7%–7.1% in Denmark, Finland, Norway, and Sweden [37] and 1.6% in Singapore [38].

Gonadal hormones were reported to affect the emotional processing in the brain [39]. Allopregnanolone, a neuroactive metabolite of progesterone, is a potent positive allosteric modulator of the γ-aminobutyric acid _A_ (GABA_A_) receptor complex that could induce or relieve anxiety symptoms [40]. In women, anxiety symptoms are related to allopregnanolone concentrations in an inverted U-shaped curve. Negative mood symptoms occur when its serum concentration is similar to that of endogenous luteal phase levels [41]. Thus, the mood symptoms of PMDD could be caused by the paradoxical effect of allopregnanolone mediated by the GABA_A_ receptor. Furthermore, women with PMDD have reduced GABA_A_ receptor sensitivity to diazepam and pregnanolone and increased sensitivity to allopregnanolone [41]. These GABA_A_ receptor characteristics of PMDD might contribute to patients’ vulnerability to anxiety symptoms [35] and their risk of GAD [23] as demonstrated in this study. However, the mechanism, in particular relating to ovarian hormones, underlying the association between GAD and PMDD should be evaluated in the future.

### 4.2. Premenstrual Change in Depression, Anxiety, and Irritability in Women with PMDD and GAD

Fundamental PMDD symptoms, such as irritability and depression, are exacerbated during the late luteal phase [42] and are resolved after menstruation each month. Our results in Table 1 present the characteristics of premenstrual exacerbation and relief in the follicular phase for these emotional symptoms. Table 3 does not present a significant difference in luteal PMDD severity, depression, or irritability between women with PMDD, with and without GAD. This result did not indicate that women with PMDD and GAD experience more severe PMDD symptoms. Although they experienced higher anxiety symptoms than those without GAD, their anxiety symptoms did not significantly increase in the luteal phase. This result did not indicate premenstrual exacerbation of anxiety symptoms among women with both PMDD and GAD. This could have occurred because their GAD anxiety symptoms were already high in the follicular phase and plateaued without increasing further in the luteal phase. However, these claims deserve further investigation.

Women with PMDD and GAD had higher PMDD severity, anxiety, depression, and irritability in the follicular phase than those without it. The PMDD severity of women with PMDD and GAD significantly decreased in the follicular phase. However, the paired *t* test revealed that their anxiety, depression and irritability did not significantly decrease in the follicular phase. Thus, persistent anxiety, depression, and irritability symptoms could contribute to their higher follicular PMDD severity. Regarding the chronicity of GAD [43], anxiety and irritability symptoms could not be reasonably relieved in the follicular phase among women with GAD. Moreover, a depressed mood was persistent in the follicular phase. The overlap among manifestations of psychopathology and the similarity in etiological models between depressive disorder and GAD [44] might explain the higher follicular depression scores in the GAD group. Because depression, irritability and anxiety are the fundamental symptoms of PMDD, their persistence in the follicular phase might indicate that their emotional symptoms were not relieved in the follicular phase. The reduced PMDD severity in the follicular phase might result from the relief of somatic symptoms such as headaches, sleep problems, appetite changes, and reduced interest. Thus, clinicians must pay more attention to anxiety, depression and irritability, not only in the luteal phase but also in the follicular phase among women with comorbid PMDD and GAD.

### 4.3. Role of Behavior Inhibition, Depression, and Irritability in Association between PMDD and GAD

Our results demonstrated that both PMDD and GAD are associated with higher BIS scores, in line with previous results [23,24]. Furthermore, irritability is a symptom of PMDD and GAD [3]. The results in Table 2 demonstrated the partially mediating effect of behavior inhibition and irritability in the association between GAD and PMDD. Furthermore, depression, a cardinal symptom of PMDD [8], completely mediated the association between GAD and PMDD based on the theory of Baron and Kenny [34]. These results suggest that the shared presentation of depression, irritability, and behavior inhibition might contribute to the association between PMDD and GAD. Thus, effective intervention or treatment for these mediators, such as serotonin-specific reuptake inhibitors [43], might attenuate the association between PMDD and GAD.

### 4.4. Limitations

There are several limitations in this study. Firstly, since many candidates of the PMDD group were excluded based on the PMDD symptomatic cycle, the number of participants was relatively limited [33]. Second, the causal relationship between GAD and PMDD could not be confirmed based on the cross-sectional research design in this presenting study. Finally, the PMDD symptoms were rated weekly rather than daily during the menstrual cycle. Thus, we could not totally prevent the memory bias and mental averaging effect.

## 5. Conclusions

This study demonstrates that women with GAD are more likely to have PMDD. Women with PMDD and GAD had more severe anxiety symptoms than those without GAD. Furthermore, their depression and irritability were not relieved in the follicular phase. Thus, GAD should be assessed among women with PMDD. Moreover, mental health professionals must intervene when persistent emotional symptoms emerge in the follicular phase. Depression, behavior inhibition, and irritability mediate the association between PMDD and GAD. Effective intervention for these mediators should be provided to women with PMDD and GAD. Further studies are necessary to prospectively investigate the effect of these interventions in attenuating comorbid GAD among women with PMDD.

## Figures and Tables

**Table 1:** Demographic data, behavior inhibition system (BIS), behavior approach system (BAS), depression, irritability, and generalized anxiety disorder in premenstrual and follicular phases among women with premenstrual dysphoric disorder (PMDD) and controls.

Variables	PMDD Group N = 100 (Mean ± SD)	Paired *t*-Test	Control Group N = 96 (Mean ± SD)	Paired *t*-Test	Independent *t*-Test
Age	24.77 ± 3.32		24.84 ± 3.46		−0.152
Educational level	16.15 ± 1.24		16.33 ± 1.26		−1.025
BIS					
Luteal	21.52 ± 2.61	3.175 **	19.85 ± 2.19	0.745	4.855 ***
Follicular	20.90 ± 2.22		19.73 ± 2.22		3.687 ***
BAS					
Luteal	41.51 ± 5.46	−1.449	40.98 ± 4.43	−0.093	0.746
Follicular	42.09 ± 4.79		41.01 ± 4.63		1.604
Depression					
Luteal	24.81 ± 8.64	4.76 ***	9.14 ± 5.77	1.73	14.99 ***
Follicular	19.15 ± 9.99		8.15 ± 5.14		9.75 ***
Irritability					
Luteal	60.40 ± 12.19	3.588 **	50.15 ± 10.94	0.768	6.189 ***
Follicular	57.35 ± 10.40		49.59 ± 11.68		4.915 ***
	N (%)		N (%)		X^2^
GAD					
Yes	14 (86.0%)		2 (14.0%)		9.28 **
No	86 (47.8%)		94 (52.2%)		

BIS: behavior inhibition in BIS/BAS scale; BAS: behavior activation in BIS/BAS scale. Irritability: Buss–Durkee Hostility Inventory-Chinese Version-Short Form score. Depression: Mandarin Chinese version of the Center for Epidemiologic Studies Depression Scale. ** *p* < 0.01; *** *p* < 0.001.

**Table 2:** Hierarchical logistic regression to evaluate the association between premenstrual dysphoric disorder and generalized anxiety disorder (GAD), after controlling for behavior inhibition or irritability.

	Wald X^2^	Df	Exp(β)	95% CI
**PMDD versus controls**				
Model 1				
Age	0.00	1	0.97	0.92–1.09
GAD	6.70	1	7.65 **	1.69–34.63
Model 2				
Age	0.02	1	1.01	0.92–1.10
GAD	4.38	1	5.22 *	1.11–24.54
Behavior Inhibition	7.69	1	1.05 **	1.02–1.09
Model 3				
Age	0.02	1	1.01	0.92–1.10
GAD	4.04	1	4.96 *	1.04–23.60
Irritability	24.26	1	1.07 ***	1.04–1.11
Model 4				
Age	0.06	1	1.02	0.89–1.15
GAD	0.79	1	2.32	0.36–14.90
Depression	54.44	1	1.28 ***	1.20–1.37

BIS: behavior inhibition in BIS/BAS scale; BAS: behavior activation in BIS/BAS scale. Irritability: Buss–Durkee Hostility Inventory-Chinese Version-Short Form score Depression: Mandarin Chinese version of the Center for Epidemiologic Studies Depression Scale * *p* < 0.05; ** *p* < 0.01; *** *p* < 0.001.

**Table 3:** Difference in premenstrual dysphoric disorder (PMDD) symptom severity, behavior inhibition system (BIS), anxiety, depression, and irritability between women with generalized anxiety disorder and those without, among women with PMDD.

Variables	GAD (Mean ± SD) N = 14	Paired *t*	Control (Mean ± SD) N = 86	Paired *t*	*t*
PMDDSQ					
Luteal	67.62 ± 18.40	2.75*	67.57 ± 19.74	13.38 ***	0.013
Follicular	46.21 ± 24.33		27.05 ± 23.25		2.841 **
BIS					
Luteal	22.07 ± 2.20	3.02*	21.43 ± 2.67	2.66 **	0.852
Follicular	21.29 ± 2.05		20.84 ± 2.25		0.698
Anxiety					
Luteal	61.43 ± 6.55	0.67	53.14 ± 10.10	4.29 ***	2.96 **
Follicular	60.50 ± 7.42		50.09 ± 2.29		3.99 ***
Depression					
Luteal	26.36 ± 5.81	0.61	24.56 ± 8.04	4.87 ***	0.72
Follicular	24.57 ± 8.04		18.27 ± 10.04		2.23 *
Irritability					
Luteal	63.29 ± 11.40	0.13	59.93 ± 12.31	3.72 ***	0.955
Follicular	63.07 ± 7.64		56.41 ± 10.53		2.265 *

BIS: behavior inhibition in BIS/BAS scale; BAS: behavior activation in BIS/BAS scale. Irritability: Buss–Durkee Hostility Inventory-Chinese Version-Short Form score Depression: Mandarin Chinese version of the Center for Epidemiologic Studies Depression Scale * *p* < 0.05; ** *p* < 0.01; *** *p* < 0.001.
